# Geographical Variation in Egg Mass and Egg Content in a Passerine Bird

**DOI:** 10.1371/journal.pone.0025360

**Published:** 2011-11-14

**Authors:** Suvi Ruuskanen, Heli Siitari, Tapio Eeva, Eugen Belskii, Antero Järvinen, Anvar Kerimov, Indrikis Krams, Juan Moreno, Chiara Morosinotto, Raivo Mänd, Erich Möstl, Markku Orell, Anna Qvarnström, Juha-Pekka Salminen, Fred Slater, Vallo Tilgar, Marcel E. Visser, Wolfgang Winkel, Herwig Zang, Toni Laaksonen

**Affiliations:** 1 Section of Ecology, Department of Biology, University of Turku, Turku, Finland; 2 Department of Biological and Environmental Sciences, University of Jyväskylä, Jyväskylä, Finland; 3 Institute of Plant and Animal Ecology, Russian Academy of Sciences, Ekaterinburg, Russia; 4 Faculty of Biological and Environmental Sciences, University of Helsinki, Helsinki, Finland; 5 Zvenigorod Biological Station of Moscow State University, Moscow, Russia; 6 Institute of Systematic Biology, Daugavpils University, Daugavpils, Latvia; 7 Departamento de Ecologıa Evolutiva, Museo Nacional de Ciencias Naturales-CSIC, Madrid, Spain; 8 Department of Zoology, University of Tartu, Tartu, Estonia; 9 University of Veterinary Medicine, Vienna, Austria; 10 Department of Biology, University of Oulu, Oulu, Finland; 11 Department of Animal Ecology, University of Uppsala, Uppsala, Sweden; 12 Laboratory of Organic Chemistry and Chemical Biology, University of Turku, Turku, Finland; 13 School of Biosciences, Cardiff University, Cardiff, United Kingdom; 14 Netherlands Institute of Ecology, Wageningen, The Netherlands; 15 Institute of Avian Research ‘Vogelwarte Helgoland’, Wilhelmshaven, Germany; 16 Goslar, Germany; 17 Finnish Museum of Natural History, University of Helsinki, Helsinki, Finland; Arizona State University, United States of America

## Abstract

Reproductive, phenotypic and life-history traits in many animal and plant taxa show geographic variation, indicating spatial variation in selection regimes. Maternal deposition to avian eggs, such as hormones, antibodies and antioxidants, critically affect development of the offspring, with long-lasting effects on the phenotype and fitness. Little is however known about large-scale geographical patterns of variation in maternal deposition to eggs. We studied geographical variation in egg components of a passerine bird, the pied flycatcher (*Ficedula hypoleuca*), by collecting samples from 16 populations and measuring egg and yolk mass, albumen lysozyme activity, yolk immunoglobulins, yolk androgens and yolk total carotenoids. We found significant variation among populations in most egg components, but ca. 90% of the variation was among individuals within populations. Population however explained 40% of the variation in carotenoid levels. In contrast to our hypothesis, we found geographical trends only in carotenoids, but not in any of the other egg components. Our results thus suggest high within-population variation and leave little scope for local adaptation and genetic differentiation in deposition of different egg components. The role of these maternally-derived resources in evolutionary change should be further investigated.

## Introduction

Phenotypes, life-histories and reproductive strategies in many animal and plant taxa show geographic variation, indicating spatial variation in selection regimes [1]–[6]. In particular, reproductive investment, in the form of clutch or egg size, shows latitudinal trends inter- and intra-specifically across taxonomic groups [7]–[10] and is particularly well studied in birds [11], [12]. A number of hypotheses have been put forward to explain the latitudinal variation in avian reproductive investment (clutch and egg size), including energy, nutrient or time limitations or variation in predation pressure [12]–[17]. In general, reproductive investment (e.g. egg size) is subject to several selection pressures: parent-offspring conflict over per-offspring investment, trade-off between egg size and number, and competing demands for resources in the parents [18]. Thus if selection optimizes offspring fitness, decreasing environmental productivity with latitude may be expected to lead to increasing investment in egg quality with latitude, to increase offspring survival [18]–[20]. Alternatively, higher adult maintenance costs in poor environmental conditions may lead to reduced investment in eggs [18], which could generate a decreasing latitudinal trend in reproductive investment.

In addition to egg size, maternal investment in offspring quality in the form of different egg components such as lipids, immune factors, hormones and antioxidants critically influences offspring development and survival in many taxa [18], [21]–[27]. Maternally-derived immunoglobulins provide the primary form of humoral immune defence for the offspring, as underdeveloped young cannot synthesize them [28]. Lysozyme enzyme destroys cell walls of bacteria and it is thus a major component of the antibacterial immunity of the egg [29]. Yolk androgens can affect offspring development and phenotype in many ways, for example growth, immunity, behaviour and plumage traits [25]. Carotenoids are antioxidants that reduce lipid peroxidation in the embryo, and they can also enhance immune function [30]. Carotenoid levels are mainly determined by their availability in the mother's diet, because they cannot be synthesized by birds or stored for a long time [30]. Deposition of several egg components is known to be affected by environmental or social conditions (e.g. food availability, parasite load or quality of mates) *within* populations [25], [30]–[34]. To our knowledge only very few studies have estimated large-scale geographical variation *among* populations in egg components (or any maternal effects) in any species (with the exception of egg size). Most of the existing studies in birds have compared deposition into eggs in two contrasting environments [35]–[40], but these results suggest that populations could differ in several maternally-derived egg components (e.g. yolk carotenoids and androgens).

Maternal effects, via maternal behavior or resource allocation, have been suggested to play an important role in trait evolution and even population differentiation [41]–[44]. Studying geographical variation in maternal deposition to eggs may be seen as the first step to reveal its evolutionary potential. As with other life-history traits, spatial variation in adaptive benefits of maternal resource allocation may lead to among-population variation in egg quality [6]. Given that deposition of several egg components affects offspring fitness and is heritable [25], [27], [45]–[47], selection on egg composition may lead to microevolution in these traits. Alternatively, among-population variation in deposition to eggs could be due to phenotypic plasticity (either due to resource limitation or adaptive resource allocation), which may even constrain genetic differentiation [48].

We studied geographical variation in egg components of the pied flycatcher (*Ficedula hypoleuca*) by collecting egg samples from 16 populations all over Europe and analysing variation in egg and yolk mass, albumen lysozyme enzyme activity, yolk immunoglobulin, yolk androgen (testosterone and androstenedione) and yolk carotenoid concentrations. The study species shows geographical variation in reproductive traits: Timing of breeding gets later and clutch size decreases towards the north, where breeding seasons are shorter and more unpredictable, temperatures are lower and food availability during egg-laying may be more limited (as egg laying begins at earlier ambient phenology) [15], [49]–[52]. Egg size has been found to increase (linear or quadratic trends) towards the north in other European passerines [53], [54], but trends in the pied flycatcher are unclear [55]. For example, investment in larger eggs may be selected in northern latitudes because of lower hatching failure of larger eggs in cold temperatures [56]. Within-population variation in egg components in relation to environmental variables in the pied flycatcher and its sister species, the collared flycatcher (*Ficedula albicollis*) has been revealed in the recent years: yolk carotenoid and immunoglobulin levels have been found to vary in relation with laying order [57], [58], timing of breeding [59], female condition [57], [59] and caterpillar availability (carotenoids) [58], [60]. Furthermore, among-clutch variation in yolk androgen levels seems to be associated with environmental factors such as timing of breeding [61], [62], food supply (Laaksonen, T. unpublished), social stimulation [59], female characteristics such as condition [47], [62], and male quality [61], [63]. Yolk androgen allocation has also been shown to be repeatable and heritable [47], [62]. However, no information on the among-population variation in egg components in the study species exists.

Our first aim was to quantify the extent of among- and within-population variation in egg mass and key egg components at a large geographical scale – a rarely studied topic. We suggest that large among-population variation may be an indicator of differential selection and genetic differences, but it can also be induced by phenotypic plasticity in these traits. Low variation among populations and large variation within populations, however, is likely to indicate little scope for genetic differences, potentially high phenotypic plasticity, or suggest that there is no spatial variation in adaptive benefits of maternal resource deposition to eggs. Alternatively, this may indicate that females are constrained in their allocation, potentially due to costs for themselves or offspring [25]. However, our analysis is exploratory and thus we cannot separate with certainty the cause of variation among populations. Secondly, we studied variation in egg components in relation to geographical location and habitat. We hypothesized that if maternal deposition to eggs improves offspring quality, and if selection leads to increasing offspring quality in marginal environments [19], [20], we should observe a latitudinal increase in egg quality in the study range. Alternatively, if resources needed for self-maintenance (nutrients or energy during egg laying) limit deposition of different egg components, an opposite pattern may emerge. However, it is not easy to determine which kind of deposition represents improved quality for individual egg components, as with respect to some egg components, high levels may even be detrimental (such as immunosuppression by yolk androgens) [25]. Different egg components may furthermore be under different constraints and selective forces. We also investigated whether deposition of egg components is affected by biotic population level factors such as timing of breeding or clutch size and whether deposition is associated with reproductive success. Finally, we studied the co-variation between egg components, to examine the hypothesis that females could simultaneously modify deposition of several egg components. For example, high levels of yolk androgens promote growth and may be immunosuppressive, which may increase offspring need of antioxidants and maternal immune factors [64]. Although our analyses are exploratory, we consider these as an important first step towards understanding variation in resource allocation and the potential role of maternal allocation to eggs in evolutionary processes.

## Methods

### Study species

**Figure 1:**
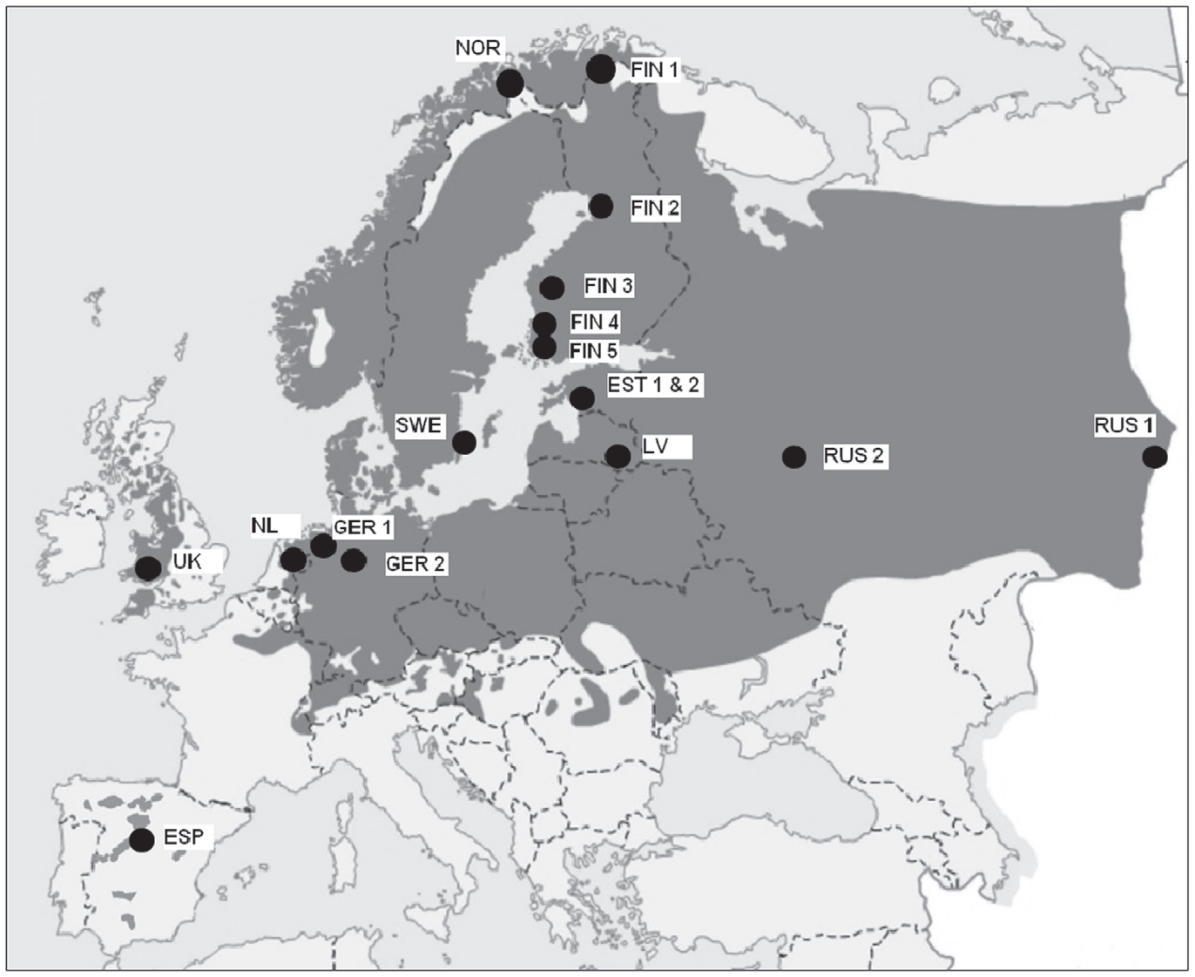
Locations of the populations sampled. Grey area illustrates breeding range of pied flycatchers in Europe (map modified from: Birds of the Western Palearctic, version 2.0.1., Oxford University press, 2003). FIN 1 = Kevo, Finland, NOR = Skibotn, Norway; FIN 2 = Oulu, Finland; FIN 3 = Kauhava, Finland; FIN 4 = Harjavalta, Finland; FIN 5 = Turku, Finland; EST, Pärnu, Estonia; SWE = Öland, Sweden; RUS 1 = Revda, Russia; LV = Kraslava, Latvia; RUS 2 = Moscow, Russia; GER 1 = Lingen, Germany; UK = Powys, United Kingdom; NL = Buunderkamp, The Netherlands; GER 2 = Harz, Germany; ESP = Lozoya, Spain.

The pied flycatcher is a small (12–13 g), migratory, insectivorous, hole-nesting forest passerine bird [65]. It breeds throughout a large range over Europe and Western Siberia (breeding range is illustrated in Fig. 1) and winters in western Africa. Pied flycatchers are single-brooded and the modal clutch size is five to six eggs. The pied flycatcher is an abundant species that easily accepts artificial nest boxes, and thus it is a common model species in studies of avian ecology and evolution.

### Field protocol

Egg samples were collected from 16 different nest-box study populations across the breeding range of the pied flycatcher during spring and summer 2007 (see Fig. 1). In each population, nest-boxes were checked at three day intervals to monitor the progress of nesting. When eggs were found in the nest, these were marked and the nest was visited the following days to collect the freshly laid third or fourth egg of each clutch. Thus the collected eggs were not incubated, and there was no embryo development. In each population, egg sampling was conducted evenly throughout the breeding season if possible (leaving out very first and last nests), to avoid bias in egg composition due to potential seasonal variation in egg components. The egg components may vary systematically with laying order [25], [33], [57], [58], [62], [66], but the middle eggs should represent the average values of the clutch. For example, in a Finnish population, the clutch mean and fourth egg yolk androgen levels were highly correlated (testosterone: r_s_ = 0.85, N = 24, p<0.001; androstenedione r_s_ = 0.78, N = 24, p<0.001). The position of the egg in the laying sequence, laying date and fresh mass (∼0.01 g, using standard scales, e.g. Pesola) were recorded. If mass could not be measured, the maximum length and width of the eggs were measured (∼0.01 mm, using digital or manual caliper) and the Hoyt volume (volume = length * breadth^2^ * 0.51) [67], which is highly correlated with the egg mass, was calculated. Hoyt volume was further converted to egg mass by multiplying the volume by 1.06 [68]. Eggs were thereafter stored at −20°C until the laboratory analyses. Nests were monitored throughout the breeding season, to record final clutch size and number of hatchlings and fledglings.

Eggs were collected in 16 populations, and from each population, ca. 20 eggs were acquired (range 4–32 eggs per population; see Table S1 for detailed sample sizes). The sampling area covers large parts of the breeding area of pied flycatchers in Europe. Locations of the sampling populations are indicated in Fig. 1 and Table S1. Data from one population (Estonia) were collected from two different habitats (coniferous and deciduous) forming a mixed mosaic of habitat patches, which differ in some breeding parameters (e.g. laying dates, R. Mänd, pers. comm.). In the analyses these two were considered as one population, but the habitat variable was different for the two habitats.

### Ethics statement

Egg collection and all animal work was conducted under relevant national and international guidelines and under licenses from environmental authorities and ethical committees in each country (UK: Countryside Council for Wales, license number OTH:SB:06:200; Sweden: Swedish National Board for Laboratory Animals and the Bird Ringing Centre of the Swedish Museum of Natural History, license number Dnr 33-07; Spain: Consejería de Medio Ambiente y Ordenación del Territorio, Comunidad de Madrid, license number 10/289334.9/07; Russia: Department of Rosprirodnadzor in Sverdlovsk oblast and Council of Zvenigorod Biological Station of Moscow State University and bio-ethic codex of Biological Faculty of MSU, license numbers N 212/647-249, protocol #04 November 16, 2005; NL: Animal Experimental Committee of the KNAW, license number DEC protocol no CTE 07-04; Latvia: Ethical Committee for Research in Ecology and Evolution, Daugavpils University, license number 4/ZOO-2009; Germany: Landkreis Goslar, Niedersachsen, Bundesrepublik Deutschland, license number Az. 66 24 03-2.3.1.1; Finland and northern Norway (Lapland): West Finland Regional Environment Centre, Environmental center of Southwest Finland, Environmental center of North Ostrobothia and Environmental center of Lapland, license numbers LSU-2006-L-509 (254), LOS-2007-L-264-254, PPO-2007-L-400-254 and LAP-2007-L-261-254; Estonia: Estonian Environmental Board, license number 1-4.1/11/100).

### Laboratory protocol

Yolk and albumen were carefully separated in the laboratory. Yolks were weighed (∼0.1 mg) and mechanically homogenised (as the distribution of e.g. hormones may vary among egg layers) [69], [70]. Approximately half of the yolk was used for androgen analysis and ¼ for both immunoglobulin and carotenoid analyses. Yolks were frozen at −20°C. All samples were analysed simultaneously and by the same laboratory, to avoid problems due to different analysis methods. Immunoglobulin and lysozyme analyses were conducted at the Department of Biological and Environmental Science, University of Jyväskylä, Finland. Carotenoid analyses were conducted at the Department of Chemistry, University of Turku, Finland. Hormone analyses were conducted at the University of Veterinary Medicine, Vienna, Austria.

### Immunoglobulin (IgG) analysis

Antibody concentrations were determined using an indirect enzyme-linked immuno-sorbent assay (ELISA). The method is as described in [71]. Briefly, the yolk samples were weighed and diluted in 1∶3 of distilled water. The samples were centrifuged at 13000 g for 15 min (at +4°C), in order to separate the clear immunoglobulin supernatant from the precipitated extra material. The supernatants were collected and diluted (1∶2000) in 1% BSA-PBS. The standard stock solution was made by pooling an equal volume (5 µl) of the supernatant of each yolk sample (N = 351) and giving the undiluted cocktail an arbitrary concentration of one million units of immunoglobulin per ml (1000000 U/ml). After the standard was frozen with glycerol (1∶1), the final concentration of the standard stock was 125 000 U/ml.

The wells were coated with 50 µl anti-chicken IgG (Sigma C-6409, whole molecule, produced in rabbit) in carbonate buffer overnight (o/n) at +4°C. Hereafter the wells were masked with 100 µl 1% BSA-PBS (1 h at room temperature, RT). Duplicates of samples and standards (50 µl) were incubated three hours in RT. The conjugated secondary antibody (Sigma A-9171, anti-chicken IgG whole molecule, alkaline phosphatase conjugated, 1∶2000 dilution) (50 µl) was added and incubated (o/n) at +4°C. After adding the substrate (100 µl) (p-nitro phenyl phosphate, Sigma 104-0 in 1 M diethanolamine buffer) the absorbances were measured at 405 nm (Multiskan Ascent, Therma Oy, Finland). The wells were washed three times with 0.05% Tween 20 in 1× PBS between the steps (first two washes 200 µl, third wash 400 µl). Inter-assay variation was 8.53% and intra-assay variation 7.56%.

### Lysozyme analysis

A micro-plate modification of the turbidimetric assay [72] was used to determine lysozyme activity as described in [73]. Shortly, albumen was diluted in phosphate buffer (67 mM, pH 6.2, dilution 1∶500). A *Micrococcus lysodeikticus* (Sigma M-3770) suspension was prepared in phosphate buffer (0.5 mg/ml). The lysozyme of the samples will start degrading the bacterial cell walls, which can be seen as clearing of the *Micrococcus* suspension and measured as change in absorbance with a microplate reader (Multiskan Ascent, Therma Oy, Finland). 100 µl of diluted albumen and 100 µl of *Micrococcus* were added to the wells on the plate and the absorbance was measured at 450 nm in room temperature for 30 min using duplicates of samples. Before each measurement, the plate was mixed for 10 s. The results are given as lysozyme activity = change in absorbance units ×1000/min (hereafter Δabs ×1000/min). The linear part of the declining curve was used to calculate the change in absorbance. Inter-assay variation was 5.6% and intra-assay variation was 2.0%.

### Androgen analysis

For measuring the concentrations yolk testosterone (T) and androstenedione (A4), we used a method similar to that described in [69], [74]. To extract steroids, after thawing, each yolk sample was suspended in 400 µl of distilled water and 1600 µl methanol and vortexed twice for 30 s. Samples were then stored overnight at 4°C. Samples were then vortexed and 1 ml of the suspension was transferred into a new vial. The suspension was then diluted with 1∶5 assaybuffer, vortexed for 30 min and stored at −20°C overnight to precipitate apolar lipids. After centrifugation (−15°C, 2500 g, 10 min) 20 µl of the supernatant were used for enzyme immunoassays. For full descriptions of antibodies and validation see [70], [74]–[76]. Inter-assay variation was 9.9% (low level pool) and 5.5% (high level pool) for testosterone and 12.9% and 9.3% for androstenedione. Intra-assay variation was 7.9% for testosterone and 10.1% for androstenedione.

### Carotenoid analysis

Yolk carotenoid concentrations were measured using a method similar to that described in [77]. For carotenoid analyses ca. 10 eggs from each population were randomly chosen (due to time and financial constrains). Egg yolk was freeze-dried (at −33°C for 48 h) and ground into fine powder. A known amount of fine powder (approx. 20 mg), was extracted three times with 100% acetone. The solvent was evaporated from the combined extract under vacuum and the residue dissolved into a small volume of 100% acetone. The carotenoid composition of the extracts was analysed with high-performance liquid chromatography at 450 nm using an YMC C-30 (250×4 mm, i.d., 5 µm) column and a gradient from 86% aqueous acetone into 97% aqueous acetone (flow rate 1.5 ml/min). β-carotene was quantified using commercial β-carotene as a standard and the other carotenoids (lutein, zeaxanthin, other xanthophylls and unidentified carotenoids) using commercial lutein as a standard. All the standards were purchased from Extrasynthese (France). Sum of all carotenoids was used in the analyses (total carotenoid concentration, µg/g). Carotenoid profiles have been described in [78].

### Population background data

In addition to data from the individual nests from which eggs were collected, background data from the study populations was collected (Table S1). This data included coordinates of the populations (latitude and longitude) and habitat type data (coniferous forest, N = 7 populations; deciduous forest, N = 6 populations; mixed forest, N = 4 populations).

### Statistical analyses

All statistical analyses were conducted with SAS 9.2. Lysozyme enzyme activity was squared and concentrations of yolk total immunoglobulin, yolk testosterone, yolk androstenedione and yolk carotenoids were log-transformed for normality. First we quantified among- and within-population variation in egg components using simple General Linear Models (GLM) in which the egg component was the response variable and population the explanatory variable. We then studied geographic variation in egg components with linear mixed models (MIXED). The independent factors in the models were: latitude, longitude, 2^nd^ order terms of latitude and longitude, habitat (coniferous, mixed and deciduous), habitat×latitude and habitat×longitude. We investigated the effect of longitude (along with latitude) on egg components as the climatic continentality gradient across Europe (affecting rainfall and temperatures) may also affect environmental variables important in determining allocation of egg components. The analyses of specific carotenoid profiles and total carotenoids are reported elsewhere [78], but the analyses of total carotenoids are presented also here for a comparison with other egg components. Population was included as a random factor to account for potential non-independence of the samples from one population. Since some of our sampling sites were closer to one another than others, we first checked whether there was spatial autocorrelation in model residuals. Moran's I coefficients ranged from −0.019 to 0.022 (N = 155 for carotenoids and 333–349 for other egg components), indicating no spatial autocorrelation, thus a default covariance structure (variance components) was used. Non-significant terms were dropped from the models one-by-one, starting from the interactions. The dropped non-significant main effects and interactions were again added in the reduced models one at a time and statistics after re-introducing them into the final model are reported. We calculated Spearman correlations among the average values of egg components and average breeding parameters (laying date, clutch size, hatching success, and fledging success) for each population (nests where eggs were collected). We further analyzed covariation among the egg components using linear mixed models (proc MIXED) by assigning each egg component as response and explanatory variable at a time, and including population as a random factor, accounting for non-independence of the samples from each population.

## Results

### Among-population vs. within-population variation in egg components

**Figure 2:**
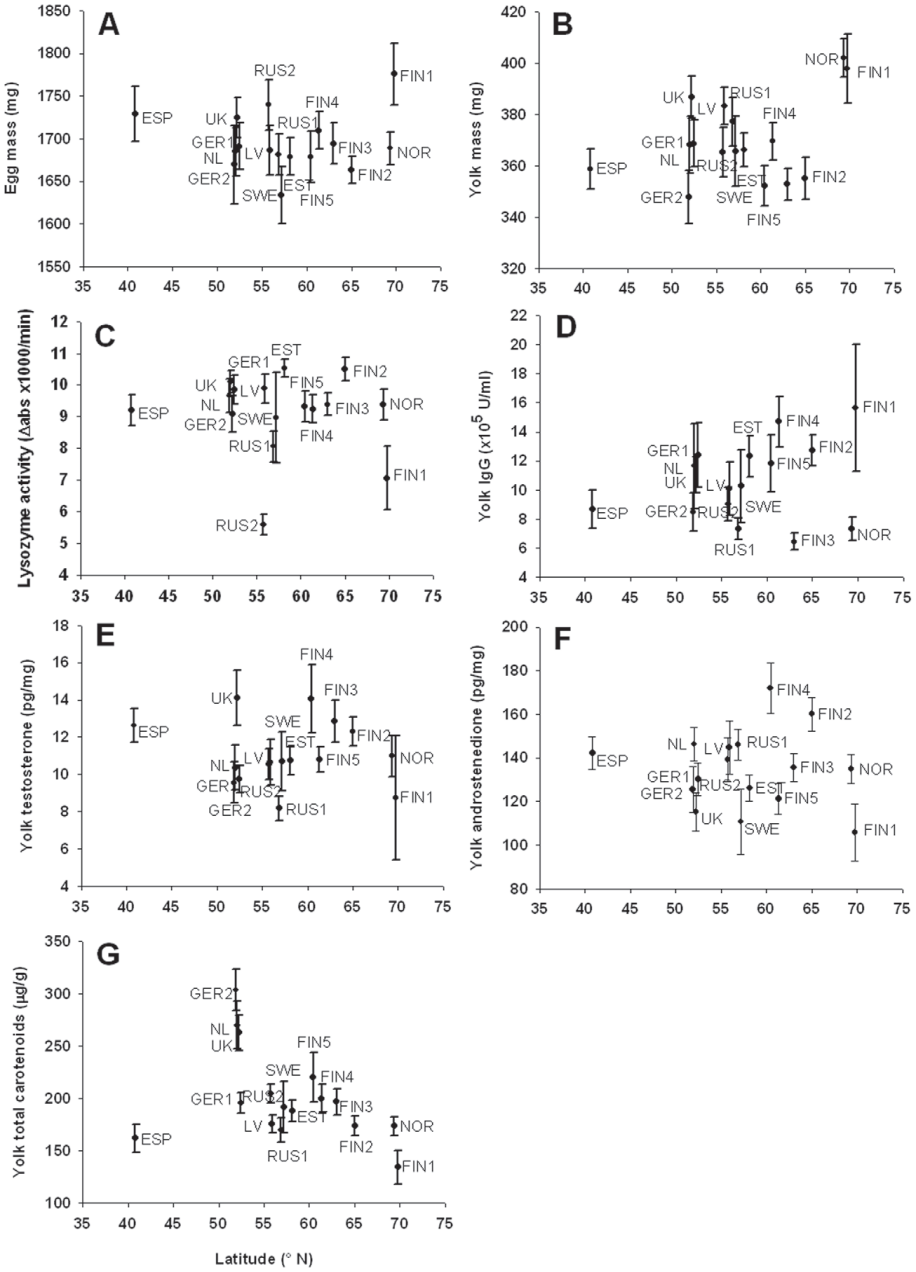
A–G. Among-population variation in the measured egg components. Among-population variation (mean ± SE) in relation to latitude in the measured egg components. Abbreviations of each country and location are the following: FIN 1 = Kevo, Finland, NOR = Skibotn, Norway; FIN 2 = Oulu, Finland; FIN 3 = Kauhava, Finland; FIN 4 = Harjavalta, Finland; FIN 5 = Turku, Finland; EST, Pärnu, Estonia; SWE = Öland, Sweden; RUS 1 = Revda, Russia; LV = Kraslava, Latvia; RUS 2 = Moscow, Russia; GER 1 = Lingen, Germany; UK = Powys, United Kingdom; NL = Buunderkamp, The Netherlands; GER 2 = Harz, Germany; ESP = Lozoya, Spain. Sample sizes are shown in Table S1.

Population averages and range of variation in the egg components in each population are shown in Figure 2A–G and Table S1. There was significant variation among populations in each egg component with the exception of egg mass (all other p-values≤0.01; Table 1). In yolk mass, albumen lysozyme activity, yolk immunoglobulin concentration, yolk testosterone and yolk androstenedione concentrations population explained around 10% of the total variation. In yolk carotenoid concentration, population explained around 40% of the total variation (Table 1). For comparison, population explained 84% of variation laying date and 12% in clutch size, respectively.

**Table 1:** Among-population (N = 16 populations) variation in egg size and egg components.

Egg component	R^2^	F	p	N
**Egg mass (g)**	0.03	0.75	0.73	342
**Yolk mass (mg)**	0.11	2.84	<0.001	349
**Albumen lysozyme activity (Δabs ×1000/min)**	0.22	6.10	<.0001	347
**Yolk immunoglobulins (U/ml)**	0.11	2.63	0.01	351
**Yolk testosterone (ng/mg)**	0.10	2.41	<0.01	351
**Yolk androstenedione (ng/mg)**	0.12	3.24	<.0001	351
**Yolk total carotenoids (µg/g)**	0.39	6.28	<.0001	162

Results are from a GLM with population as the explanatory variable.

### Geographic variation in egg components

There were no geographic trends (either linear or quadratic) in any of the egg components, with the exception of carotenoids (Table 2). Total carotenoid concentration showed a quadratic trend with latitude (β ± SE = −0.00079±0.0002, Table 2, Fig. 2G): Carotenoid concentration appeared to be low in the most southerly population (Spain), highest in Central Europe (i.e. 50–55°N) and decreased again towards the north. Total carotenoid concentration showed also a linear decrease trend from west to east (β ± SE = −0.003±0.0013, Table 2, see also ref 78 for patterns in carotenoid composition and profiles). None of the egg components differed among habitats (Table 2).

**Table 2:** Geographical variation in egg size and egg components.

	Egg mass		Yolk mass		Squared lysozyme		Log IgG		Log T		Log A4		Log carotenoids	
Expl. variables	*DDF*	*F*	*DDF*	*F*	*DDF*	*F*	*DDF*	*F*	*DDF*	*F*	*DDF*	*F*	*DDF*	*F*
Latitude	17.1	2.1	13.8	1.32	14.2	0.04	15	0.25	12.5	0.08	12.7	0.02	**12.9**	**10.59^a^**
Longitude	17.3	1.08	12.7	0.15	13.4	2.83	14.3	0.45	11.1	3.68	12	1.03	**12**	**6.73^b^**
Quadratic latitude	14	1.18	13.5	0.64	13.3	0.03	13.9	0.08	11	0.55	11.8	0.1	**13.1**	**11.2^c^**
Quadratic longitude	11.2	1.24	11.5	0.25	12.0	0.75	12.8	0.67	9.74	0.19	10.9	0.03	10.7	0.51
Habitat	22.5	1.22	19	0.09	24.9	0.16	21.7	0.32	16.4	0.99	18.2	0.51	16.8	2.28
Latitude × habitat	25.4	1.39	20.9	0.11	25.2	0.25	13.1	2.21	17.3	0.55	18.8	0.53	10.5	1.97
Longitude × habitat	4.01	1.53	20.8	0.01	25.8	0.36	13	0.71	17.8	0.22	20.3	0.14	8.92	0.14

Results are from linear mixed models explaining geographical variation in egg components. Population was included as a random effect in all models. The only significant effects are indicated with bold and letters (a–c). IgG = yolk immunoglobulins, A4 = yolk androstenedione, T = yolk testosterone.Numerator df is 2 for habitat and 1 for other explanatory variables. a: p = 0.006, b: p = 0.023, c: p = 0.005.

### Correlations among egg components and breeding parameters at the population level

Averages of breeding parameters in the nests where eggs were collected are presented in Table S1. We found that laying date was weakly negatively correlated with carotenoid concentration (r_s_ = −0.43, p = 0.08, N = 17) but not with any of the other egg components (−0.32<r_s_<0.13, p>0.30, N = 17). Clutch size was positively correlated with testosterone levels (r_s_ = 0.5, p = 0.03, N = 16) but not with any other egg component (−0.12<r_s_<0.12, p>0.33, N = 16). None of the egg components was correlated with hatching success (−0.40<r_s_<0.15, p>0.08, N = 14). Lysozyme activity and immunoglobulin concentration were negatively correlated with fledging success (lysozyme: r_s_ = −0.63, p = 0.02 and IgG: r_s_ = −0.79, p = 0.0013, respectively, N = 13 in both). Fledging success was not correlated with any other egg component (−0.37<r_s_<0.37, p>0.20, N = 13).

### Correlations among egg components

Egg mass and yolk mass were positively correlated across the populations (Table 3), as well as testosterone and androstenedione concentrations (Table 3). Lysozyme activity was weakly negatively correlated with egg mass (Table 3). There were no other significant correlations between the measured egg components (Table 3).

**Table 3:** Covariation among egg components.

	Yolk mass	Lysozyme	IgG	T	A4	Carotenoids
**Egg mass**	1.82±0.14	−0.35±0.17	28.56±23.51	12.46±15.05	16.54±22.32	25.31±86.36
	F_1, 336_ = 162.12***	F_1, 339_ = 4.37*	F_1 ,254_ = 1.30	F_1, 310_ = 0.69	F_1, 276_ = 0.55	F_1, 154_ = 0.09
**Yolk mass**		−0.11±0.056	5.51±7.41	6.51±4.66	5.80±7.00	−11.24±25.87
		F_1,330_ = 3.7	F_1, 347_ = 0.55	F_1, 346_ = 1.96	F_1, 346_ = 0.69	F_1, 116_ = 0.19
**Lysozyme**			6.12±7.03	7.46±4.47	11.49±6.70	−25.86±27.7
			F_1, 340_ = 0.76	F_1, 341_ = 2.80	F_1, 342_ = 2.90	F _1,150_ = 0.87
**IgG**				−0.01±0.04	−0.01±0.05	0.01±0.22
				F_1, 349_ = 0.19	F_1,348_ = 0.07	F_1, 123_ = 0.00
**T**					0.61±0.07	0.38±0.36
					F_1, 349_ = 70.41***	F_1, 101_ = 1.10
**A4**						−0.04±0.23
						F _1, 142_ = 0.03

***<0.001, **<0.01, *<0.05.

Regression coefficients (± SE) and F-values among the measured egg components across whole data. Population was included as a random factor to control for non-independence of the samples from each population. IgG = yolk immunoglobulins, T = yolk testosterone, A4 = yolk androstenedione.

## Discussion

We found that there was significant variation among pied flycatcher populations in all the measured egg components, with the exception of egg mass. However, population explained only a small part of the total variation in the egg components (with the exception of carotenoids). Thus the majority of variation was found among individuals within populations. We found no geographical trends in any egg components, with the exception of carotenoids.

The low among-population variation found for the egg components may indicate that the benefits of differential allocation do not differ among environments and that there is no strong local selection on the allocation of resources to eggs, despite large environmental differences. A recent genetic analysis also revealed that pied flycatcher populations, especially in the Northern and Eastern Europe, are not differentiated from each other, suggesting extensive gene flow and little scope for local adaptations [79]. Thus the potential for egg components to play a role in population divergence in offspring traits may not be strong, although it has been shown for some other maternal effects [37], [42], [44], [80]. However, there are also alternative pathways which could facilitate the role of maternal effects via eggs in trait evolution and population divergence. Thus populations may differ in within-clutch patterns of deposition [81] or in relationships between egg components.

An exception for the general pattern of low among-population variation was carotenoids, in which the among-population variation was considerably higher (40%) than in other egg components. Carotenoid concentrations appear to have only a minor genetic component [46], [82], and their levels in the yolk are mainly determined by carotenoid availability in the mother's diet [58], [60], [83]. The large among-population variation is therefore likely mainly due to variation in carotenoid availability in different environments or differences in their absorption or transfer to yolk. Thus variation in carotenoid levels most likely reflects phenotypic (resource-dependent) rather than genetic variation. Yolk carotenoid levels have been found to vary among populations also in other species [36], [58], [60].

What explains the extensive within-population variation in resource deposition to eggs? Previous studies indicate that deposition of several egg components is associated with environmental or social conditions within populations: For example, deposition of androgens in the study species as well as in other species has been associated with timing of breeding [61], [62], food availability (Laaksonen, T. unpublished), breeding density or female or male quality [25], [47], [59], [61]–[63]. Deposition of antibodies and antibacterial enzymes has been associated with timing of breeding [59], parasite load, female condition and mate quality [34], [57], [59], [84]. Thus it may be that variation in the relevant environmental factors (especially female and mate quality) affecting deposition of egg components is simply larger within than among populations. Furthermore, differences in reproductive physiology or genetic factors among individuals within populations may play a role [45], [58]–[63]. Egg size and deposition of several egg components (yolk mass, yolk immunoglobulins, yolk testosterone, potentially lysozyme) indeed have been found to have a genetic component [27], [45]–[47]. However, as this is the first time intra-specific variation in maternal effects in eggs has been studied on a large scale, more studies quantifying among- and within-population variation are needed to reveal patterns in other species and the evolutionary potential of maternal effects in eggs.

In contrast to our hypothesis, egg quality, measured in the form of different egg components, did not generally vary geographically, despite the large latitudinal changes in for example temperature during egg-laying, duration of the breeding season, predictability of environmental conditions and potentially also in food supply. This may imply that deposition of different egg components is simply not reflecting increasing egg quality (*sensu* life-history theory), and benefits of differential allocation may not differ among populations. Alternatively, even if higher resource deposition would be beneficial for the offspring, females may be constrained in their deposition to eggs, due to costs for themselves [18], [25], [45]. We may also speculate that perhaps the relevant environmental and social factors (e.g. female and mate quality, timing of breeding, breeding densities and parasite exposure, see above) that are found to affect deposition of egg components in the study species [59]–[63], do not show geographical trends, but vary more within than among populations. We further found that, at the population level, timing of breeding, clutch size or hatching success did not seem to be associated with deposition of the egg components. Only carotenoids (total carotenoids and the proportion of lutein and other xantophylls), showed geographical trends (either linear or quadratic), decreasing from Central Europe towards the north [78]. This most likely reflects the availability of carotenoid-rich food (especially lepidopteran larvae), which is lower in north due to northern populations starting egg-laying earlier relative to the tree phenology than central European populations [78]. None of the egg components, with the exception of specific carotenoids (lutein and other xanthophylls) [78] varied among different habitats, although variation in for example food quality and quantity among habitats has been shown [85], [86].

In contrast to our hypothesis, we found generally no correlations among egg components across the whole data set. Only egg mass and yolk mass as well as the two androgen hormones were strongly correlated, as expected on the basis of previous studies [45], [47]. Albumen lysozyme enzyme activity was negatively correlated with egg mass, a result supported by some previous studies [87], suggesting a potential cost or constraint in allocating lysozyme to eggs. Our results are also consistent with earlier studies [64], [88] showing that individual egg level or clutch level correlations between egg mass, antioxidants, immunoglobulins and androgens were not found despite of correlated within-clutch variation. The lack of correlation among these compounds may simply reflect that they are regulated by different processes. However, co-variation among egg components may differ in different environments, for example due to differential trade-offs in allocation between self and offspring in relation to food availability and condition or prevalence of infectious diseases. This issue should be further investigated.

### Conclusions

We found that there was significant variation among populations in most egg components, but most of the variation was among individuals within populations, probably due to high plasticity in deposition to eggs. No geographic patterns in egg quality, with the exception of carotenoids, were found. As our study was exploratory, more studies are needed to thoroughly understand the role of maternal effects in evolutionary change.

## Supporting Information

Table S1 Among-population variation in breeding parameters and egg components in the pied flycatcher. Mean (± SD), minimum and maximum values of the measured egg components for each study population are shown along with the locations of the study populations, habitat types and averages of breeding parameters of the nests where eggs were collected. Lat = latitude (°N), Long = longitude (°E), N = sample size per population. For laying date 1 = 1.4.2007.
(DOC)
