# Supplementary material for: Geographical Variation in Egg Mass and Egg Content in a Passerine Bird
Source: PLoS One. 2011 Nov 14;6(11):e25360. doi: 10.1371/journal.pone.0025360 (PMC3215694; doi:10.1371/journal.pone.0025360)
Supplement: Table S1 — Among-population variation in breeding parameters and egg components in the pied flycatcher. Mean (± SD), minimum and maximum values of the measured egg components for each study population are shown along with the locations of the study populations, habitat types and averages of breeding parameters of the nests where eggs were collected. Lat = latitude (°N), Long = longitude (°E), N = sample size per population. For laying date 1 = 1.4.2007. (DOC) [file pone.0025360.s001.doc]

Table S1.

| **Country** | **Area** | **Lat** | **Long** | **Habitat** | **Laying date** | **Clutch size** | **Hatching success** | **Fledging success** | **N** | **Egg mass (g)** | | **Yolk mass (mg)** | |
| --- | --- | --- | --- | --- | --- | --- | --- | --- | --- | --- | --- | --- | --- |
|  |  |  |  |  | mean | mean | mean | mean |  | mean ± SD | min - max | mean ± SD | min - max |
| Finland (1) | Kevo | 69 | 27 | deciduous | 71.0 | . | . | . | 4 | 1.78 ± 0.07 | 1.70 - 1.87 | 397.8 ± 27.1 | 370.4 - 434.3 |
| Norway | Skibotn | 69 | 21 | coniferous | 62.9 | 6.62 | 91.1 | 98.2 | 21 | 1.69 ± 0.09 | 1.55 - 1.89 | 402.0 ± 33.9 | 333.6 - 458.1 |
| Finland (2) | Oulu | 65 | 25 | mixed | 57.5 | 6.37 | 63.5 |  | 22 | 1.66 ± 0.07 | 1.54 - 1.80 | 355.1 ± 38.2 | 299.9 - 481.5 |
| Finland (3) | Kauhava | 63 | 23 | coniferous | 58.4 | 6.72 | 79.2 | 90.0 | 32 | 1.69 ± 0.13 | 1.41 - 2.00 | 352.7 ± 34.7 | 292.5 - 444.4 |
| Finland (4) | Harjavalta | 61 | 22 | coniferous | 57.1 | 6.41 | 99.1 | 80.1 | 32 | 1.71 ± 0.12 | 1.48 - 1.95 | 369.5 ± 40.6 | 296.9 - 458.3 |
| Finland (5) | Turku | 60 | 22 | deciduous | 58.0 | 6.47 | 96.4 | 85.7 | 21 | 1.68 ± 0.14 | 1.36 - 1.91 | 352.1 ± 34.7 | 293.5 - 420.7 |
| Estonia (1)* | Pärnu | 58 | 25 | coniferous | 49.6 | 6.85 | 97.3 | 84.5 | 21 | 1.68 ± 0.12 | 1.41 - 1.90 | 362.4 ± 42.1 | 272.9 - 439.5 |
| Estonia (2)* | Pärnu | 58 | 25 | deciduous | 50.8 | 6.43 | 84.6 | 55.5 | 21 | 1.68 ± 0.16 | 1.30 - 1.97 | 369.8 ± 44.1 | 268.0 - 444.6 |
| Sweden | Öland | 57 | 17 | mixed | 52.7 | 6.30 | 75.0 | 92.5 | 10 | 1.63 ± 0.06 | 1.60 - 1.70 | 365.7 ± 41.1 | 311.2 - 431.0 |
| Russia (1) | Revda | 57 | 60 | mixed | 57.6 | 6.32 | 93.2 | 92.1 | 22 | 1.68 ± 0.11 | 1.49 - 1.89 | 377.1 ± 44.8 | 297.4 - 513.7 |
| Latvia | Kraslava | 56 | 27 | coniferous | 52.5 | 6.00 | 95.1 | 88.1 | 21 | 1.69 ± 0.13 | 1.23 - 1.87 | 383.3 ± 33.9 | 315.4 - 447.1 |
| Russia (2) | Moscow | 56 | 37 | coniferous | 52.4 | 6.15 | . | . | 22 | 1.74 ± 0.14 | 1.39 - 1.96 | 365.2 ± 45.5 | 296.1 - 494.1 |
| Germany (1) | Lingen | 52 | 7 | coniferous | 30.6 | 6.06 | 96.7 | 76.2 | 22 | 1.69 ± 0.13 | 1.46 - 1.88 | 368.6 ± 43.0 | 302.7 - 457.5 |
| UK | Powys | 52 | -3 | deciduous | 36.4 | 6.90 | 86.9 | 97.0 | 20 | 1.72 ± 0.11 | 1.54 - 1.99 | 386.7 ± 36.3 | 330.6 - 484.0 |
| Netherlands | Buunderkamp | 52 | 6 | mixed | 33.5 | 6.27 | 91.3 | 84.9 | 15 | 1.68 ± 0.11 | 1.48 - 1.82 | 368.2 ± 43.4 | 290.4 - 459.6 |
| Germany (2) | Harz | 52 | 11 | deciduous | 36.9 | 6.17 | . | . | 18 | 1.67 ± 0.19 | 1.35 - 2.05 | 347.9 ± 43.8 | 269.6 - 427.2 |
| Spain | Lozoya | 41 | -4 | deciduous | 49.6 | 5.85 | 84.9 | 100.0 | 27 | 1.73 ± 0.17 | 1.46 - 2.08 | 358.8 ± 40.8 | 295.0 - 475.7 |

| **Country** | **Area** | **Albumen lysozyme (∆abs x1000/min)** | | **Yolk IgG (x10^5 U/ml)** | | **Yolk T (pg/mg)** | | **Yolk A4 (pg/mg)** | | **Yolk carotenoids (µg/g)** | |
| --- | --- | --- | --- | --- | --- | --- | --- | --- | --- | --- | --- |
|  |  | mean ± SD | min - max | mean ± SD | min - max | mean ± SD | min - max | mean ± SD | min - max | mean ± SD | min - max |
| Finland (1) | Kevo | 7.1 ± 2.0 | 4.7 - 9.6 | 15.6 ± 8.7 | 8.6 - 26.8 | 8.8 ± 6.7 | 2.5 - 15.9 | 105.8 ± 25.9 | 71.7 - 134.6 | 134.1 ± 27.6 | 102.3 - 150.6 |
| Norway | Skibotn | 9.4 ± 2.2 | 3.1 - 12.0 | 7.3 ± 3.7 | 1.8 - 15.9 | 11.0 ± 5.0 | 3.0 - 24.6 | 134.8 ± 30.0 | 106.1 - 203.7 | 173.4 ± 28.0 | 126.1 - 224.0 |
| Finland (2) | Oulu | 10.5 ± 1.8 | 7.2 - 13.8 | 12.7 ± 5.0 | 3.2 - 21.1 | 12.3 ± 3.6 | 5.6 - 19.1 | 160.2 ± 35.9 | 109.2 - 214.8 | 173.5 ± 29.4 | 140.1 - 229.5 |
| Finland (3) | Kauhava | 9.4 ± 2.0 | 5.3 - 12.2 | 6.4 ± 3.3 | 1.8 - 15.1 | 12.8 ± 6.4 | 4.8 - 34.8 | 135.6 ± 36.3 | 58.1 - 201.8 | 196.6 ± 39.4 | 147.3 - 269.0 |
| Finland (4) | Harjavalta | 9.2 ± 2.5 | 3.3 - 13.0 | 14.7 ± 9.9 | 1.8 - 41.9 | 10.8 ± 3.9 | 4.5 - 18.4 | 121.2 ± 40.9 | 67.5 - 255.9 | 199.6 ± 44.3 | 142.9 - 257.7 |
| Finland (5) | Turku | 9.3 ± 2.2 | 3.8 - 12.9 | 11.8 ± 9.0 | 2.1 - 37.8 | 14.0 ± 8.3 | 4.5 - 42.9 | 171.9 ± 53.0 | 89.8 - 280.5 | 219.8 ± 75.4 | 122.6 - 341.9 |
| Estonia (1)* | Pärnu | 10.7 ± 1.7 | 7.6 - 13.4 | 11.5 ± 9.4 | 2.1 - 42.2 | 10.7 ± 5.1 | 5.7 - 25.4 | 130.1 ± 43.1 | 40.5 - 218.6 | 166.4 ± 38.2 | 104.4 - 210.8 |
| Estonia (2)* | Pärnu | 10.3 ± 1.9 | 5.9 - 13.2 | 13.1 ± 9.0 | 1.3 - 26.5 | 10.8 ± 5.0 | 3.2 - 22.0 | 122.1 ± 35.3 | 70.6 - 192.1 | 209.4 ± 41.8 | 132.9 - 282.5 |
| Sweden | Öland | 9.0 ± 3.5 | 2.1 - 12.0 | 10.3 ± 7.9 | 2.0 - 31.0 | 10.7 ± 5.1 | 2.0 - 15.4 | 110.8 ± 47.5 | 54.8 - 205.2 | 191.4 ± 73.0 | 88.4 - 321.1 |
| Russia (1) | Revda | 8.1 ± 2.3 | 4.6 - 12.7 | 7.3 ± 3.5 | 1.4 - 15.8 | 8.2 ± 3.0 | 4.3 - 16.2 | 146.2 ± 33.3 | 71.3 - 215.7 | 169.8 ± 36.5 | 107.0 - 232.6 |
| Latvia | Kraslava | 9.9 ± 2.1 | 5.2 - 12.6 | 10.1 ± 8.3 | 1.2 - 32.7 | 10.6 ± 5.6 | 4.1 - 28.9 | 144.8 ± 56.6 | 79.7 - 274.4 | 175.6 ± 25.7 | 139.3 - 218.8 |
| Russia (2) | Moscow | 5.6 ± 1.5 | 3.7 - 10.4 | 9.0 ± 5.3 | 2.2 - 22.4 | 10.5 ± 3.8 | 4.5 - 18.3 | 139.4 ± 47.1 | 66.0 - 227.8 | 204.5 ± 27.2 | 156.8 - 247.3 |
| Germany (1) | Lingen | 9.9 ± 2.1 | 4.3 - 12.7 | 12.4 ± 10.5 | 1.2 - 52.9 | 9.7 ± 3.5 | 4.2 - 21.5 | 130.2 ± 34.8 | 57.9 - 192.1 | 195.4 ± 31.2 | 150.0 - 243.7 |
| UK | Powys | 9.1 ± 2.6 | 3.7 - 12.1 | 11.0 ± 5.6 | 3.0 - 23.4 | 14.1 ± 6.6 | 6.6 - 36.5 | 115.3 ± 39.1 | 61.7 - 212.0 | 262.6 ± 54.5 | 176.4 - 387.0 |
| Netherlands | Buunderkamp | 10.1 ± 1.3 | 7.7 - 12.6 | 11.7 ± 11.3 | 2.8 - 47.5 | 10.4 ± 4.7 | 0.6 - 19.9 | 148.9 ± 28.7 | 90.8 - 194.5 | 269.6 ± 71.7 | 143.8 - 369.8 |
| Germany (2) | Harz | 9.7 ± 2.2 | 4.2 - 13.0 | 8.5 ± 5.6 | 2.4 - 24.2 | 9.6 ± 4.6 | 1.9 - 22.1 | 125.4 ± 44.8 | 61.1 - 257.6 | 303.6 ± 61.9 | 223.1 - 424.0 |
| Spain | Lozoya | 9.2 ± 2.5 | 3.7 - 13.1 | 8.7 ± 6.9 | 3.2 - 38.5 | 12.6 ± 4.6 | 4.7 - 24.3 | 142.3 ± 39.4 | 62.3 - 220.7 | 161.8 ± 42.6 | 102.1 - 235.5 |

*Data from Estonia were collected from two different habitats (1 = coniferous, 2 = deciduous), which differ in some breeding traits (R. Mänd, pers. comm.). In the analyses these two were considered as one population.
